# Serum Metalloproteinases 2 and 9 as Predictors of Gait Status, Pressure Ulcer and Mortality after Hip Fracture

**DOI:** 10.1371/journal.pone.0057424

**Published:** 2013-02-20

**Authors:** David N. Gumieiro, Bruna P. M. Rafacho, Andrea F. Gonçalves, Priscila P. Santos, Paula S. Azevedo, Leonardo A. M. Zornoff, Gilberto J. C. Pereira, Luiz S. Matsubara, Sergio A. R. Paiva, Marcos F. Minicucci

**Affiliations:** 1 Surgery and Orthopedic Department, Botucatu Medical School, UNESP - Univ Estadual Paulista, Botucatu, Brazil; 2 Internal Medicine Department, Botucatu Medical School, UNESP – Univ Estadual Paulista, Botucatu, Brazil; University of Bochum, Germany

## Abstract

**Introduction:**

The aim of this study is to evaluate the serum activity of metalloproteinases (MMPs) -2 and -9 as predictors of pressure ulcer (PU), gait status and mortality 6 months after hip fracture.

**Methods:**

Eighty-seven patients over the age of 65 admitted to the orthopedic unit from January to December 2010 with hip fracture were prospectively evaluated. Upon admission, patient demographic information, including age, gender and concomitant diseases, was recorded. Blood samples were taken for analysis of MMP -2 and -9 activity by gel zymography and for biochemical examination within the first 72 hours of the patient’s admission, after clinical stabilization. The fracture pattern (neck, trochanteric or subtrochanteric), time from admission to surgery, surgery duration and length of hospital stay were also recorded.

**Results:**

Two patients were excluded due to the presence of pathological fractures (related to cancer), and three patients were excluded due to the presence of PU before admission. Eighty-two patients, with a mean age of 80.4 ± 7.3 years, were included in the analysis. Among these patients, 75.6% were female, 59.8% had PU, and 13.4% died 6 months after hip fracture. All patients underwent hip fracture repair. In a univariate analysis, there were no differences in serum MMP activity between hip fracture patients with or without PU. In addition, the multiple logistic regression analysis models, which were adjusted by age, gender, length of hospital stay and C-reactive protein, showed that the pro-MMP-9 complexed with neutrophil gelatinase-associated lipocalin form (130 kDa) was associated with gait status recovery 6 months after hip fracture.

**Conclusions:**

In conclusion, serum pro-MMP-9 is a predictor of gait status recovery 6 months after hip fracture.

## Introduction

The incidence of hip fractures has been rising in recent years, and it will most likely continue to increase due to an aging population [1]–[3]. According to Hu et al., 1.5 million hip fractures occur annually worldwide, and this number may reach 4.5 million in 2050 [4].

Pressure ulcer (PU) is a frequent complication of hip fracture, with an incidence of 8.8% to 55%. It has a major impact on the cost of hospital care, quality of life, and mortality [5]. However, the adoption of a care recommendation standard for PUs did not reduce their incidence[6]. Healing of PUs normally occurs in a predictable sequence of phases that ends with scar formation. These processes are regulated by numerous molecules, including growth factors, cytokines, proteinases, and the inhibitors of these molecules [7]. Some studies that analyze wound fluids and biopsies collected from PUs showed that the presence of excessive concentrations of activated forms of matrix metalloproteinases (MMP) -2 and MMP-9 might impede the healing process. These data suggest that these proteinases could destroy growth factors, receptors and extracellular proteins essential for PUs healing [7]–[8].

The MMPs are a family of more than 25 species of zinc-dependent proteases that are essential for normal tissue remodeling and are involved in a number of pathological conditions such as cancer, inflammatory and cardiovascular diseases[9]. These enzymes are synthesized as inactive zymogens and are secreted in the extracellular matrix as proenzymes of pro-MMPs, which remain quiescent until the propeptide domain is cleaved. The activity of MMPs is controlled by the action of specific MMPs inhibitors or TIMPs[9].

These proteinases also participate in bone remodeling and in fracture healing[10]. Delays in bone healing or even nonunion of the bones could be related to the concentrations of MMPs or the behavior of these enzymes over time. Henle et al. studied serum concentrations of MMPs and TIMPs during normal and delayed fracture healing[11]. They showed that systemic MMP and TIMP concentrations could be a reflection of local enzyme regulatory mechanisms during fracture healing. In addition, an increased MMP/TIMP ratio was associated with the pathophysiological processes leading to fracture nonunion [11]. However, the association between the serum activity of MPPs -2 and -9, PU development, gait status and the mortality in hip fracture patients has not yet been established.

Thus, the aim of this study is to evaluate the serum activity of MMPs -2 and -9 as predictors of PU, gait status and mortality 6 months after hip fracture.

## Materials and Methods

This study was approved by the Ethics Committee of the Botucatu Medical School of Medicine. Written informed consent was obtained from all patients. Eighty-seven consecutive patients over the age of 65 admitted to the orthopedic unit with hip fractures from January to December 2010 were prospectively evaluated. The presence of a pathological hip fracture and a PU before hospital admission was the exclusion criterion. All patients were treated according to specific protocols depending on the type of fracture.

The number of the patients needed using t test to achieve 80% power was 84. The sample size was calculated based on the MMP-9 values in hypertension patients. It is import to note that we could not found any MMP results in hip fracture patients [12].

Upon admission, patient demographic information, including age, gender and concomitant diseases, was recorded. Blood samples were taken for analysis of MMP -2 and -9 activity and biochemical examination within the first 72 hours of the patient’s admission, after clinical stabilization. The fracture pattern (neck, trochanteric or subtrochanteric), time from admission to surgery, surgery duration and length of hospital stay were also recorded.

All patients were followed for 6 months after the fracture. The presence of PU, gait status, and mortality were recorded. These outcomes were evaluated by two examiners (orthopedists) with expertise in this issue on the first day after surgery, at hospital discharge and at 15, 45, 90 and 180 days after hospital discharge. For the patients who died before 6 months after discharge, we considered PU and gait status evaluation at the last report. Patients were classified according to gait status as ambulators (patients who walk with or without help = 0) or non-ambulators (patients who could not walk = 1).

A PU was defined as an injury to the skin or underlying tissue over a bony prominence in any of the four stages defined by the National Pressure Ulcer Advisory Panel [13]. Patients were considered to have diabetes or hypertension if they were using medication chronically for these diseases. Cardiovascular diseases were considered when patients had stroke, coronary heart disease, peripheral artery disease or heart failure.

### Laboratory Analysis

Total serum levels of C-reactive protein (CRP), albumin, glucose, creatinine and urea were measured using the dry chemistry method (Ortho-Clinical Diagnostics VITROS 950®, Johnson & Johnson).

### Zymography

Serum MMP activity was determined as reported by Tyagi et al [14]. In brief, 2 μg of serum were diluted in application sample buffer consisting of 0.5 M Tris, pH 6.8, 100% glycerol, and 0.05% bromophenol blue. The samples were loaded into the wells of 8% SDS-polyacrylamide containing 1% gelatin. Electrophoresis was carried out in a Bio-Rad apparatus at 80 V for 2 hours, until the bromophenol blue reaches the bottom of the gel. The gel was removed and washed 2 times with 2.5% Triton-X-100 and then washed with 50 mM Tris pH 8.4. The gel was then incubated at 37°C overnight in activation solution consisting of 50 mMTris pH 8.4, 5 mM CaCl_2_ and Zn Cl_2_. The staining was performed for 2 hours with 0.5% coomassie blue, and destaining was performed in 30% methanol and 10% acetic acid until clear bands over a dark background were observed. Staining and destaining were performed at room temperature on a rotatory shaker. The gels were photographed, and the intensity of gelatinolytic action (clear bands) was analyzed in UVP, UV, and a White Darkhon image analyzer.

### Statistical Methods

Data are expressed as the mean ± SD or the median (including the lower and upper quartiles). Comparisons between groups for continuous variables were performed using Student’s t-test (normal distribution) or Mann-Whitney U-test (non-normal distribution). Fisher’s test or the χ^2^ test was used for all categorical data. Logistic regression was used to predict the presence of PU and gait status, and Cox regression model was used to predict mortality 6 months after hip fracture. The MMPs were tested as independent variables and adjusted by age, gender, CRP and length of hospital stay (LOS). Except for age, all independent variables in the regression models were included as continuous variables. These variables were chosen considering their clinically important significance for pressure ulcer, gait status and mortality [15]–[19].

Data analysis was performed using SigmaPlot software for Windows v12.0 (Systat Software Inc., San Jose, CA, USA). The significance level was considered to be 5%.

## Results

Eighty-seven patients were evaluated. Two patients were excluded due to the presence of pathological fractures (related to cancer), and three patients were excluded due to the presence of PU before admission. Eighty-two patients, with a mean age of 80.4 ± 7.3 years, were included in the analysis. Among these patients, 75.6% were female, 59.8% had PU, and 13.4% died 6 months after hip fracture. All patients underwent hip fracture repair.

Considering PU development as the outcome, the demographic and clinical data are presented in Table? 1. The majority of patients had trochanteric fractures (53.7%), and the fracture type did not influence PU development. The clinical features did not influence PU development. Laboratory data and MMP activity are presented in Table? 2. There were no differences between hip fracture patients with or without PU.

**Table 1:** Demographic and clinical data of 82 patients with hip fracture.

Variables	Pressure Ulcer		P value
	No (n = 33)	Yes (n = 49)	
Age (yrs)	79.0 (71.0–84.0)	83.0 (77.0–85.3)	0.06
Female, % (n^o^)	75.8 (25)	75.8 (37)	0.81
Hypertension, % (n^o^)	60.6 (20)	55.1 (27)	0.79
Diabetes, % (n^o^)	24.2 (8)	24.5 (12)	0.80
Cardiovascular disease, % (n^o^)	30.3 (10)	36.7 (18)	0.72
Statins, % (n^o^)	15.1 (5)	4.1 (2)	0.11
Fracture type, % (n^o^)			
Femoral neck	36.4 (12)	40.8 (20)	0.84
Trochanteric	54.5 (18)	53.1 (26)	
Subtrochanteric	9.1 (3)	6.1 (3)	
LOS, (days)	7.0 (4.8–9.0)	8.0 (6.0–11.3)	0.06
A-S time, (days)	5.0 (3.8–7.0)	6.0 (4.0–8.3)	0.10
Mortality, % (n°)	6.1 (2)	18.4 (9)	0.19

LOS: length of hospital stay; A-S time: admission to surgery time. Data are expressed as median (including the lower and upper quartiles).

**Table 2:** Baseline biochemical and zymography results of 82 patients with hip fracture.

Variables	Pressure Ulcer		P value
	No (n = 33)	Yes (n = 49)	
CRP, (mg/dL)	4.8 (3.3–6.9)	5.4 (3.6–12.4)	0.32
Creatinine, (mg/dL)	0.8 (0.7–1.1)	0.8 (0.7–1.1)	0.46
Urea, (mg/dL)	58.0 (36.8–75.5)	55.7 (38.8–72.4)	0.88
Glucose, (mg/dL)	111.5 (95.5–136.5)	122.5 (93.0–149.5)	0.35
Albumin, (g/L)	3.2 ± 0.6	3.2 ± 0.5	0.97
225 kDa (homodimer pro-MMP 9), (%)	21.3 (19.8–26.0)	24.9 (19.8–26.4)	0.26
130 kDa (pro-MMP 9 +NGAL), (%)	8.5 ± 3.3	8.8 ± 3.9	0.67
92 kDa (pro-MMP 9), (%)	51.1 ± 8.2	52.4 ± 7.1	0.44
72 kDa (pro-MMP 2), (%)	15.8 (11.3–21.3)	11.7 (9.6–8.3)	0.07

CRP: C-reactive protein, MMP: matrix metalloproteinase; NGAL: neutrophil gelatinase-associated lipocalin. Data are expressed as the mean ± standard deviation or median (including the lower and upper quartiles).

Considering mortality as the outcome, creatinine and urea, accessed at baseline, were higher in patients who died 6 months after hip fracture (Tables? 3 and 4).

**Table 3:** Demographic and clinical data of 82 patients with hip fracture.

Variables	Mortality in 6 months		P value
	No (n = 71)	Yes (n = 11)	
Age (yrs)	80.0 ± 7.39	82.5 ± 6.8	0.29
Female, % (n^o^)	76.1 (54)	72.7 (8)	1.00
Hypertension, % (n^o^)	57.7 (41)	54.5 (6)	1.00
Diabetes, % (n^o^)	23.9 (17)	27.3 (3)	1.00
Cardiovascular disease, % (n^o^)	35.2 (25)	27.2 (3)	0.74
Statins, % (n^o^)	9.9 (7)	0 (0)	0.59
Fracture type, % (n^o^)			
Femoral neck	38.0 (27)	45.5 (5)	0.59
Trochanteric	53.5 (38)	54.5 (6)	
Subtrochanteric	8.5 (6)	0 (0)	
LOS, (days)	8.0 (6.0–10.8)	6.0 (4.0–7.0)	0.07
A-S time, (days)	6.0 (4.0–8.0)	4.0 (3.0–6.0)	0.05

LOS: length of hospital stay; A-S time: admission to surgery time. Data are expressed as the mean ± standard deviation or median (including the lower and upper quartiles).

**Table 4:** Baseline biochemical and zymography results of 82 patients with hip fracture.

Variables	Mortality in 6 months		P value
	No (n = 71)	Yes (n = 11)	
CRP, (mg/dL)	5.2 (3.5–8.0)	4.6 (3.5–15.8)	0.78
Creatinine, (mg/dL)	0.8 (0.7–1.0)	1.2 (0.8–1.8)	0.04
Urea, (mg/dL)	53.0 (36.3–69.8)	73.6 (52.0–107.3)	0.03
Glucose, (mg/dL)	121.0 (96.0–141.8)	101.0 (88.5–172.3)	0.68
Albumin, (g/L)	3.2 ± 0.5	3.1 ± 0.5	0.65
225 kDa (homodimer pro-MMP 9), (%)	23.8 (19.9–26.5)	24.0 (19.7–26.1)	0.95
130 kDa (pro-MMP 9 +NGAL), (%)	8.9 ± 3.8	7.5 ± 2.4	0.23
92 kDa (pro-MMP 9), (%)	51.7 ± 7.7	53.0 ± 6.7	0.61
72 kDa (pro-MMP 2), (%)	13.6 (10.3–18.5)	12.7 (10.6–20.9)	0.73

CRP: C-reactive protein, MMP: matrix metalloproteinase; NGAL: neutrophil gelatinase-associated lipocalin. Data are expressed as the mean ± standard deviation or median (including the lower and upper quartiles).

A representative zymogram of serum samples is shown in Figure? 1. Gelatin zymograms of serum samples showed all forms of MMPs usually found in human, including the homodimer of the pro-MMP-9 form (225 kDa), the pro-MMP-9 complexed with neutrophil gelatinase-associated lipocalin form (130 kDa), the pro-MMP-9 form (92 kDa) and the pro-MMP-2 (72 kDa) form.

**Figure 1:**
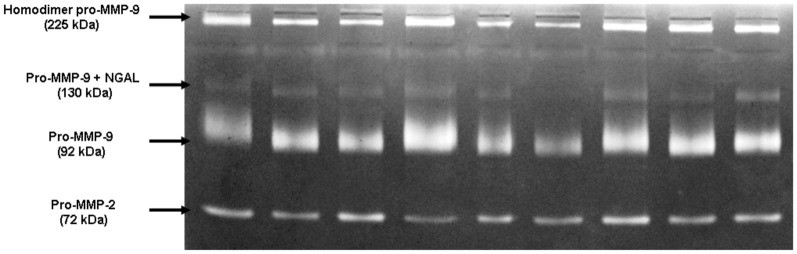
Gelatin zymograms of serum samples with all forms of MMPs usually found in human: the homodimer of the pro-MMP-9 form (225 kDa), the pro-MMP-9 complexed with neutrophil gelatinase-associated lipocalin form (130 kDa), the pro-MMP-9 form (92 kDa) and the pro-MMP-2 (72 kDa) form.

In the logistic regression and Cox regression analysis, serum MMP-2 and MMP-9 activity were not associated with PU and mortality 6 months after hip fracture (Table? 5 and 6), even when they were adjusted for age, gender, LOS and CRP. On the other hand, the pro-MMP-9 complexed with neutrophil gelatinase-associated lipocalin form (130 kDa) was a predictor of gait status recovery 6 months after hip fracture. Each 1 unit increase of pro-MMP-9 increased the chance of gait recovery by 21%. (Table? 7)

**Table 5:** Logistic regression for prediction of pressure ulcer 6 months after hip fracture.

	Odds Ratio	95% Confidence Interval	P value
225 kDa (homodimer pro-MMP 9)	1.02	0.94 – 1.10	0.63
130 kDa (pro-MMP 9 +NGAL)	1.03	0.91 – 1.16	0.66
92 kDa (pro-MMP 9)	1.02	0.96 – 1.09	0.44
72 kDa (pro-MMP 2)	0.96	0.91 – 1.02	0.15
225 kDa (homodimer pro-MMP 9)*	1.02	0.94 – 1.11	0.59
130 kDa (pro-MMP 9 +NGAL)*	1.05	0.91 – 1.21	0.51
92 kDa (pro-MMP 9)*	1.02	0.96 – 1.09	0.46
72 kDa (pro-MMP 2)*	0.95	0.89 – 1.02	0.14

MMP: matrix metalloproteinase; NGAL: neutrophil gelatinase-associated lipocalin

* Adjusted by age, gender, length of hospital stay and C-reactive protein.

**Table 6:** Cox regression models for prediction of mortality 6 months after hip fracture.

	Hazard Ratio	95% Confidence Interval	P value
225 kDa (homodimer pro-MMP 9)	0.99	0.89 – 1.10	0.10
130 kDa (pro-MMP 9 +NGAL)	0.90	0.76 – 1.07	0.23
92 kDa (pro-MMP 9)	1.02	0.94 – 1.11	0.61
72 kDa (pro-MMP 2)	1.01	0.93 – 1.08	0.85
225 kDa (homodimer pro-MMP 9)*	0.98	0.87 – 1.09	0.66
130 kDa (pro-MMP 9 +NGAL)*	0.85	0.71 – 1.03	0.10
92 kDa (pro-MMP 9)*	1.03	0.93 – 1.14	0.14
72 kDa (pro-MMP 2)*	1.04	0.94 – 1.14	0.44

MMP: matrix metalloproteinase; NGAL: neutrophil gelatinase-associated lipocalin

* Adjusted by age, gender, length of hospital stay and C-reactive protein.

**Table 7:** Logistic regression for prediction of gait status recovery 6 months after hip fracture.

	Odds Ratio	95% Confidence Interval	P value
225 kDa (homodimer pro-MMP 9)	1.03	0.94 – 1.12	0.53
130 kDa (pro-MMP 9 +NGAL)	1.14	0.99 – 1.31	0.07
92 kDa (pro-MMP 9)	0.98	0.92 – 1.04	0.55
72 kDa (pro-MMP 2)	0.98	0.92 – 1.04	0.45
225 kDa (homodimer pro-MMP 9)*	1.03	0.94 – 1.12	0.55
130 kDa (pro-MMP 9 +NGAL)*	1.21	1.03 – 1.43	0.02
92 kDa (pro-MMP 9)*	0.97	0.90 – 1.04	0.34
72 kDa (pro-MMP 2)*	0.98	0.92 – 1.05	0.52

MMP: matrix metalloproteinase; NGAL: neutrophil gelatinase-associated lipocalin

* Adjusted by age, gender, length of hospital stay and C-reactive protein.

## Discussion

The aim of this study was to evaluate the serum activity of MMPs -2 and -9 as predictors of pressure ulcer, gait status and mortality 6 months after hip fracture. Our data showed that the pro-MMP-9 was associated with gait status recovery 6 months after hip fracture. On the other hand, serum MMP-2 and MMP-9 were not associated with PU and mortality in hip fracture patients.

It is important to recognize that PU is a frequent complication after hip fracture, despite efforts employed to reduce its incidence. In our study, up to 60% of the patients had this complication within 6 months after hip fracture. The healing of PUs depends on a sequence of four phases, which are hemostasis, inflammation, proliferation, and remodeling. For a wound to heal successfully, these phases and their biophysiological functions must occur in the proper sequence and at a specific time [20].

There are local and systemic factors that influence PU healing. Among the local factors, the most important are tissue oxygenation, infection and excess production of proteinases [20]. Some studies have shown that MMPs were elevated in acute wound fluids and that MMP concentrations decreased 2 weeks after the ulcers began to heal [21]–[22]. A separate study that analyzed wound fluids and biopsies collected from PUs showed that elevated concentrations of MMP -2 and MMP-9 may influence the healing process [7]. Thus, local increases in the concentrations of MMPs could predict PU healing times. Serum concentrations of MMPs were also studied in the wound healing process. Utz et al. showed that elevated serum concentrations of MMP-2 and -7 were associated with delayed traumatic wound healing [23].

These data suggest that local MMP concentration and serum MMP concentration influence wound healing, although there is no data regarding the activity of these proteinases in ulcer development. This issue is extremely important after bone fractures because the concentrations of local and serum MMPs also increase in these situations. Henle et al. showed that serum MMPs were increased following fractures and that an increased serum MMP/TIMP ratio was associated with alterations in fracture healing [11]. Other studies also showed that high levels of serum MMPs were related with loosening of total hip replacement implants [24]–[25]. In addition, a recent study suggests that urinary levels of MMP-9 and MMP-13 may have potential as metabolic markers to monitor the progression of fracture healing [26].

Therefore, considering that the concentrations of MMPs might influence wound healing, we hypothesized that serum MMP concentrations might be associated with gait status. In accordance with this concept, our data showed that higher pro-MMP-9 concentration was a predictor of gait status recovery. This phenomenon can be explained by the decreased transformation from the inactive form to the active form of MMP-9.

Taking into account the relationship between MMP and wound healing, we also hypothesized that serum MMP concentrations would be associated with PU. Importantly, the identification of predictors of PU creates an opportunity to prevent its development. However, in our study, serum concentrations of MMP -2 and -9 were not predictors of PU development. This result could be due to the lack of the effect of these proteinases on previously intact skin.

Serum MMP levels were also associated with mortality in patients with cancer, sepsis and cardiovascular diseases [27]–[29]. The positive correlation between MMP activity and the inflammatory response is one of the explanations for the increased mortality observed in patients with high serum concentrations of MMPs. However, the role of these proteinases in mortality prediction had not yet been studied in patients with hip fractures. Our study did not show any influence of MMP-2 and MMP-9 activity on mortality prediction 6 months after hip fracture.

Finally, we should considerer the major limitations of this study. The concentration of TIMP was not measured in the serum samples of our patients, so we could not determine a ratio between collagen degradation and deposition. In addition, our study included a small sample size and patients from a unique medical center.

In conclusion, our data suggested that serum pro-MMP-9 is a predictor of gait status recovery 6 months after hip fracture.
